# The role of dysbiotic gut mycobiota in modulating risk for abdominal aortic aneurysm

**DOI:** 10.1128/spectrum.01776-24

**Published:** 2024-09-24

**Authors:** Guixiang Yao, Xinjie Zhang, Tongxue Zhang, Jiajia Jin, Zihan Qin, Xiaoyu Ren, Xiaowei Wang, Shucui Zhang, Xianlun Yin, Zhenyu Tian, Yun Zhang, Jingyong Zhang, Zhe Wang, Qunye Zhang

**Affiliations:** 1 State Key Laboratory for Innovation and Transformation of Luobing Theory, Key Laboratory of Cardiovascular Remodeling and Function Research, Chinese Ministry of Education, Chinese National Health Commission and Chinese Academy of Medical Sciences, Department of Cardiology, Qilu Hospital of Shandong University, Jinan, China; 2 Department of Biology, University College London, London, United Kingdom; 3 Department of Endocrinology & Geriatrics, Shandong Provincial Hospital, Shandong University, Jinan, China; 4 Department of Geriatrics, Shandong Provincial Hospital Affiliated to Shandong First Medical University, Jinan, China; 5 Department of Vascular Surgery, Shandong Provincial Hospital Affiliated to Shandong First Medical University, Jinan, China; Jilin University, Changchun, China

**Keywords:** gut fungi, metagenome, abdominal aortic aneurysm (AAA), *Saccharomyces cerevisiae*, gut mycobiome

## Abstract

Abdominal aortic aneurysm (AAA) is a large-vessel disease with high mortality, characterized by complex pathogenic mechanisms. Current therapeutic approaches remain insufficient to halt its progression. Fungi are important members of the gut microbiota. However, their characteristic alterations and roles in AAA remain unclear. This study investigated the role of gut fungal communities in the development of AAA through metagenomic sequencing of fecal samples from 31 healthy individuals and 33 AAA patients. We observed significant dysbiosis in the gut mycobiomes of AAA patients compared to healthy individuals, characterized by an increase in pathogenic fungi like *Candida* species and a decrease in beneficial yeasts such as *Saccharomyces cerevisiae*. The changes in fungal populations correlated strongly with clinical indicators of AAA, highlighting their potential for diagnosing and predicting AAA progression. Furthermore, our animal experiments demonstrated that *Saccharomyces cerevisiae* significantly ameliorated pathological alterations in AAA mice, suggesting a protective role for specific yeast strains against AAA development. These findings underscore the significant impact of gut mycobiomes on AAA and suggest that modulating these fungal communities could offer a novel therapeutic approach. Our research advances the understanding of the influence of gut microbiome on vascular diseases and suggests potential non-surgical approaches for managing AAA. By elucidating the diagnostic and therapeutic potential of gut fungi in AAA, this study provided important clues for future clinical strategies and therapeutic developments in the field of vascular medicine.

**IMPORTANCE:**

Our research highlights the crucial role of gut fungi in abdominal aortic aneurysm (AAA) development. By analyzing fecal samples from AAA patients and healthy controls, we discovered significant dysbiosis in gut fungal communities, characterized by an increase in harmful *Candida* species and a decrease in beneficial yeasts like *Saccharomyces cerevisiae*. This dysbiosis was correlated with the severity of AAA. Importantly, in animal experiments, supplementing with *Saccharomyces cerevisiae* significantly slowed AAA progression. These findings suggest that modulating gut fungi may offer a novel, non-surgical approach to the diagnosis and treatment of AAA, potentially reducing the need for invasive procedures.

## INTRODUCTION

Abdominal aortic aneurysm (AAA) is a severe cardiovascular disease characterized by the localized dilation of the abdominal aorta, exceeding 50% of the normal diameter (1). AAA is responsible for approximately 200,000 deaths globally each year and is a leading cause of mortality in the elderly population in some countries, particularly in males over 65 years of age (2). The asymptomatic nature of AAA in its early stages often leads to it being overlooked until the risk of rupture becomes imminent. Upon rupture, the mortality rate of AAA exceeds 80%, highlighting its lethal potential (3). Current diagnostic approaches for AAA rely primarily on imaging techniques, such as echocardiography, computed tomography (CT), and magnetic resonance imaging (MRI), with ultrasound being the preferred screening tool. However, ultrasound is limited by operator skills and patient body habitus (4). AAA treatment primarily involves surgical interventions, such as endovascular aneurysm repair (EVAR), which, while minimally invasive, requires lifelong monitoring and does not halt disease progression (5, 6). These limitations in diagnosis and treatment underscore the urgent need for novel AAA diagnostic tools and therapeutic strategies.

The pathogenesis of AAA is very complex. The cornerstone of AAA development is the degradation of the extracellular matrix (ECM) of the arterial wall, mainly driven by the overactivity of matrix metalloproteinases (MMPs), especially MMP2 and MMP9 (7). These enzymes degrade essential collagen and elastin fibers, leading to thinning and dilation of the arterial wall (8). The phenotypic transformation and apoptosis of vascular smooth muscle cells (VSMCs) further weaken the mechanical integrity of the vessel wall, promoting aneurysm formation (8). In addition, hemodynamic alterations such as abnormal shear stress, along with genetic and environmental factors including smoking and hypertension, significantly contribute to AAA development (9, 10). Clearly, our understanding of AAA pathogenesis remains incomplete, necessitating further research to elucidate the intricate mechanisms involved.

Recent research has increasingly recognized the pivotal role of the gut microbiome in various disease processes, particularly the fungal component. Despite their numerical minority compared to bacteria, gut fungi are essential for maintaining host health and modulating disease states. The intestinal mycobiome, primarily composed of yeasts and molds, constitutes 0.1%–1% of the total microbial population. It influences nutrient absorption and metabolism through unique metabolic pathways and modulates the host immune system (11–13). Emerging evidence suggests that dysbiosis of the gut mycobiome is associated with the pathogenesis of various diseases, including autoimmune and metabolic disorders. In patients with inflammatory bowel disease (IBD), an overgrowth of certain fungal species, such as *Candida tropicalis*, is commonly observed and positively correlates with the severity of intestinal inflammation (14). Furthermore, research has demonstrated increased gut fungal diversity in patients with systemic lupus erythematosus (SLE), particularly an elevated abundance of *Candida*, which may be associated with the immunoregulatory abnormalities characteristic of this disease (15). Furthermore, in patients with liver cirrhosis, gut fungal dysbiosis, characterized by increased levels of *Candida* and *Aspergillus* genera, has been linked to cognitive decline and hepatic disease progression, potentially affecting liver health by enhancing intestinal permeability and systemic inflammatory responses (16). In addition, studies have shown that obese individuals exhibit reduced gut fungal diversity, with alterations in fungal population composition correlating with host metabolic status. Gut fungi may indirectly influence obesity development by affecting energy balance and inflammatory states (17). Despite these advancements, alterations of the gut mycobiome in AAA remain unexplored, highlighting an important gap in our understanding of the potential role of intestinal fungi in this cardiovascular disease.

Gut fungal transplantation experiments have provided compelling evidence supporting a causal link between the gut mycobiome and disease states. For instance, transplantation of gut fungi from healthy individuals into mice with chronic liver disease significantly improves hepatic function by modulating immune responses and metabolic pathways (18). Similarly, the transplantation of gut fungi from obese individuals into mice induces metabolic abnormalities (19). Transferring the gut mycobiome from patients with IBD to mice elicits gastrointestinal inflammatory responses that mimic human pathology (20). Preliminary studies also indicate that the metabolites produced by certain fungal species may directly affect VSMCs, impacting their survival and function or compromising intestinal barrier integrity, thereby exacerbating arterial inflammation (21, 22). However, the specific role of gut fungi in AAA and their potential etiological contribution remains unclear. Further research is also needed to elucidate the mechanisms by which the gut mycobiome influences AAA development and progression.

In the present study, we conducted metagenomic sequencing on fecal samples from 31 healthy individuals and 33 AAA patients and successfully constructed a comprehensive map of the human gut mycobiome. Our results showed marked dysbiosis in the gut fungal communities of AAA patients, including a significant imbalance in the ratio of pathogenic fungi (such as *Candida* species) to non-pathogenic yeasts, which was closely associated with the clinical features of AAA. Furthermore, supplementation with *Saccharomyces cerevisiae* in mouse models slowed AAA progression, indicating that modulating the gut mycobiome could offer novel therapeutic strategies for AAA. These findings deepen our understanding of the relationship between gut microbiota and major diseases and provide important clues for developing microbiome-based interventions.

## RESULTS

### An atlas construction of the gut fungi using metagenomic sequencing

**Fig 1:**
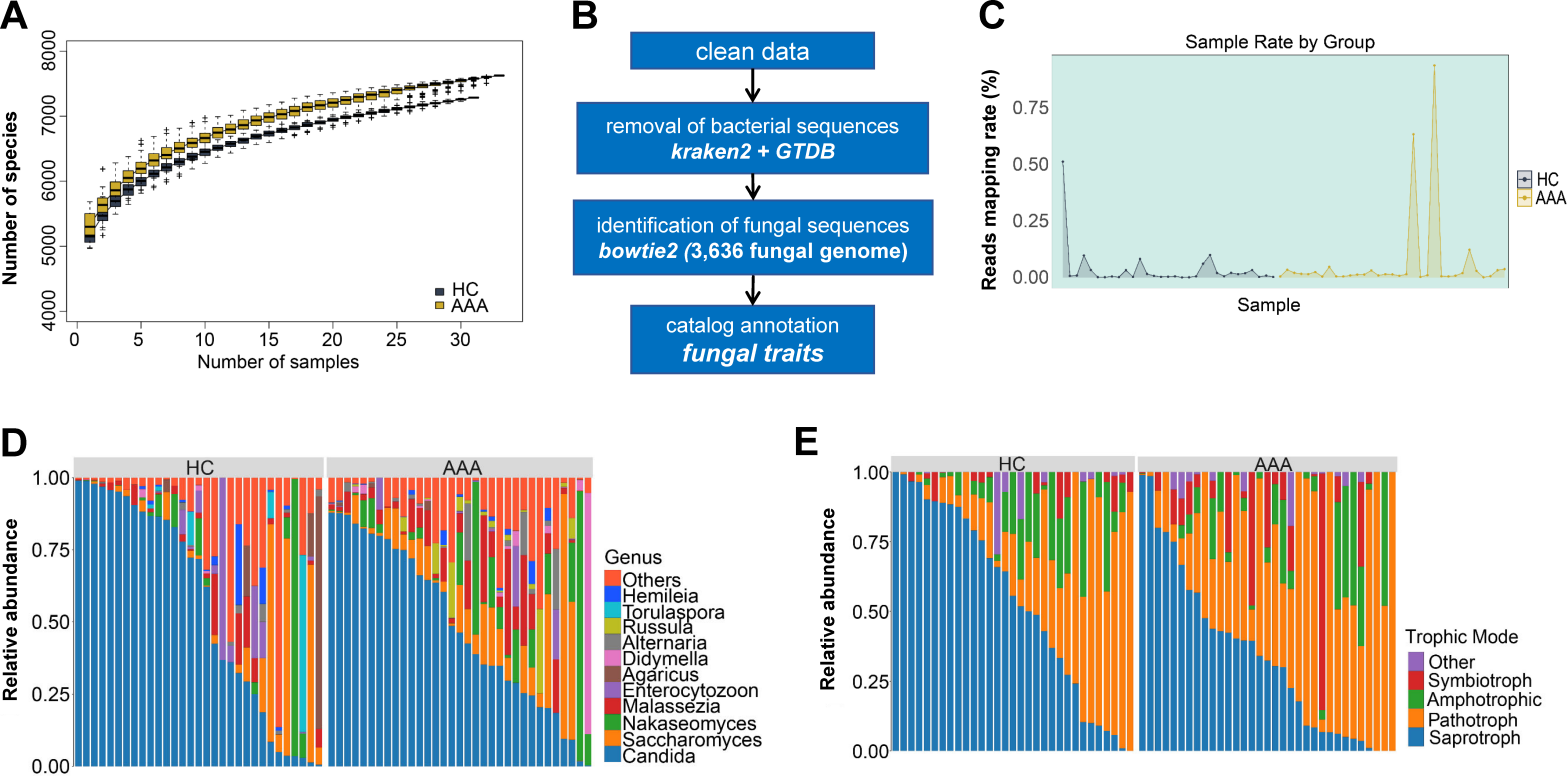
Characteristics of gut mycobiome in healthy individuals and AAA patients. (**A**) Sequencing depth rarefaction curves of samples from the healthy individuals (HC, *n* = 31) and AAA patients (AAA, *n* = 33). (**B**) Flowchart illustrating fungal composition identification based on metagenomic data. (**C**) Mapping rates of metagenomic sequencing data (reads) of each sample aligned to fungal genomes. (**D**) Stacked bar chart showing the relative abundance of gut fungi at the genus level in HC (*n* = 31) and AAA (*n* = 33) samples. (**E**) Stacked bar chart showing the relative abundance of fungi with different trophic modes in HC (*n* = 31) and AAA (*n* = 33) samples.

We performed metagenomic sequencing on fecal samples from 31 healthy individuals and 33 AAA patients (Table S1). The sequencing depth of both groups was saturated, indicating adequate genome coverage (Fig. 1A). To construct an atlas of human gut fungi, we first used Kraken2 to remove bacterial reads, and then mapped fungal sequences to an index constructed from 3,636 fungal reference genomes using Bowtie2 (Fig. 1B). The results showed that the mapping rates for fungal reads varied greatly among samples, with an overall rate of approximately 0.05% (Fig. 1C). Notably, although there was substantial inter-individual variability in the composition of gut fungi, the genera *Saccharomyces* and *Candida* were consistently the most abundant in all samples, followed by *Malassezia* and *Nakaseomyces* (Fig. 1D). In addition, we further classified the fungi according to their trophic mode, and the results showed that saprotroph and pathotroph were the main trophic modes of gut fungi (Fig. 1E), consistent with previous reports (23, 24).

### Significant dysbiosis of the gut mycobiome in AAA patients

**Fig 2:**
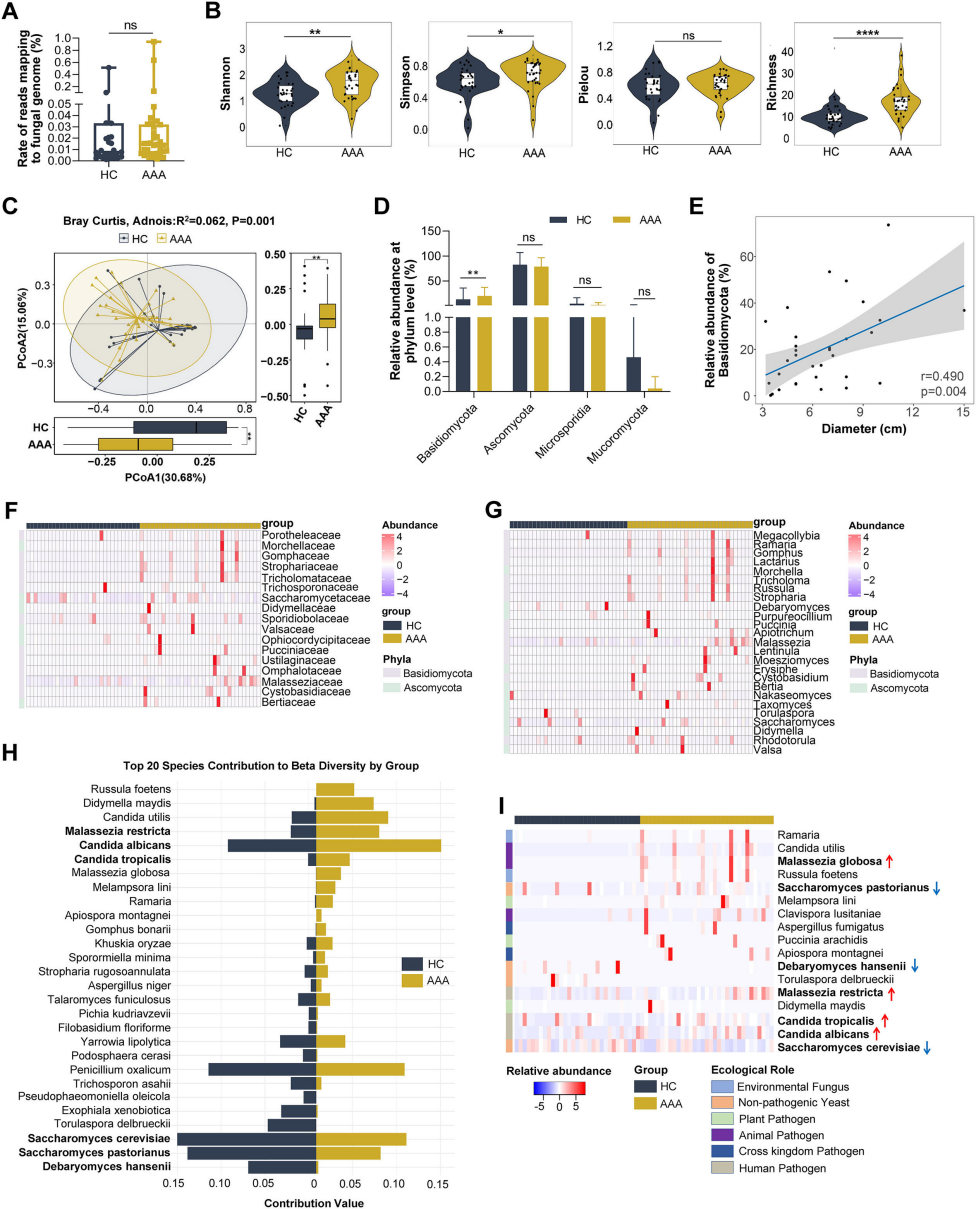
Alterations in the diversity and abundance and ecological roles of gut fungi in AAA patients. (**A**) Comparison of the mapping rate of metagenomic sequencing reads aligned to fungal genomes between healthy individuals (HC, *n* = 31) and AAA patients (AAA, *n* = 33). (**B, C**) Comparison of α-diversity indices (**B**) and β-diversity (**C**) of gut fungi between the HC (*n* = 31) and AAA (*n* = 33) groups. (**D**) Relative abundance of fungal phyla in HC (*n* = 31) and AAA (*n* = 33) groups. (**E**) Correlation between the relative abundance of *Basidiomycota* and AAA diameter. (**F, G**) Heatmap illustrating the relative abundance of gut fungi exhibiting significant differences between HC and AAA groups at the family (**F**) and genus (**G**) levels. (**H**) Distribution of species contribution to beta diversity (SCBD) of the top 20 gut fungi contributing to gut fungi β-diversity in HC and AAA groups. (**I**) Heatmap of relative abundances of gut fungal species with different ecological roles in HC and AAA groups. Blue (red) arrows indicate decreased (increased) abundance in the intestines of AAA patients. Two-tailed Wilcoxon rank-sum test was used for A and B; two-tailed Wilcoxon rank-sum test with Benjamini-Hochberg correction for multiple comparisons was used for C and D. *P < 0.05; **P < 0.01; ****P < 0.0001; ns: not significant.

We found that there were no significant differences in the mapping rates to fungal genomic reads between healthy individuals (HC) and AAA patients (AAA) (Fig. 2A). Diversity analysis showed that the Shannon, Simpson, and Richness indices of the gut fungal community were significantly increased in AAA patients. The Pielou index also showed an increasing trend but there was no significant difference (Fig. 2B). Moreover, β diversity analysis demonstrated a significant separation in the species composition between the two groups (Fig. 2C). These findings indicated that the diversity and composition of gut fungal species were significantly altered in AAA patients.

To identify AAA-specific species with differential abundance, we performed a differential analysis at several taxonomic levels of fungi. At the phylum level, we found that the abundance of *Basidiomycota* was significantly increased in the gut of AAA patients, which was significantly positively correlated with a key clinical indicator, the diameter of AAA. No significant changes were observed at other phylum levels (Fig. 2D and E). At the family and genus levels, we found that the most differentially abundant fungi in the gut of AAA patients were mainly those with increased abundance, primarily belonging to the *Basidiomycota* phylum (Fig. 2F and G). At the species level, we identified species that significantly contributed to the observed differences in β diversity between healthy individuals and AAA patients. These included *Debaryomyces hansenii*, *Saccharomyces pastorianus*, and *Saccharomyces cerevisiae* in the HC group, and *Malassezia restricta*, *Candida albicans*, and *Candida tropicalis* in the AAA group (Fig. 2H). Further differential analysis at the species level showed significant differences in the abundance of these fungal species between the two groups. They were mainly pathogenic fungi from *Candida* and *Malassezia*, as well as non-pathogenic yeasts from *Saccharomyces* and *Debaryomyces* (Fig. 2I).

### Disrupted fungi-bacteria symbiotic homeostasis in the gut of AAA patients

**Fig 3:**
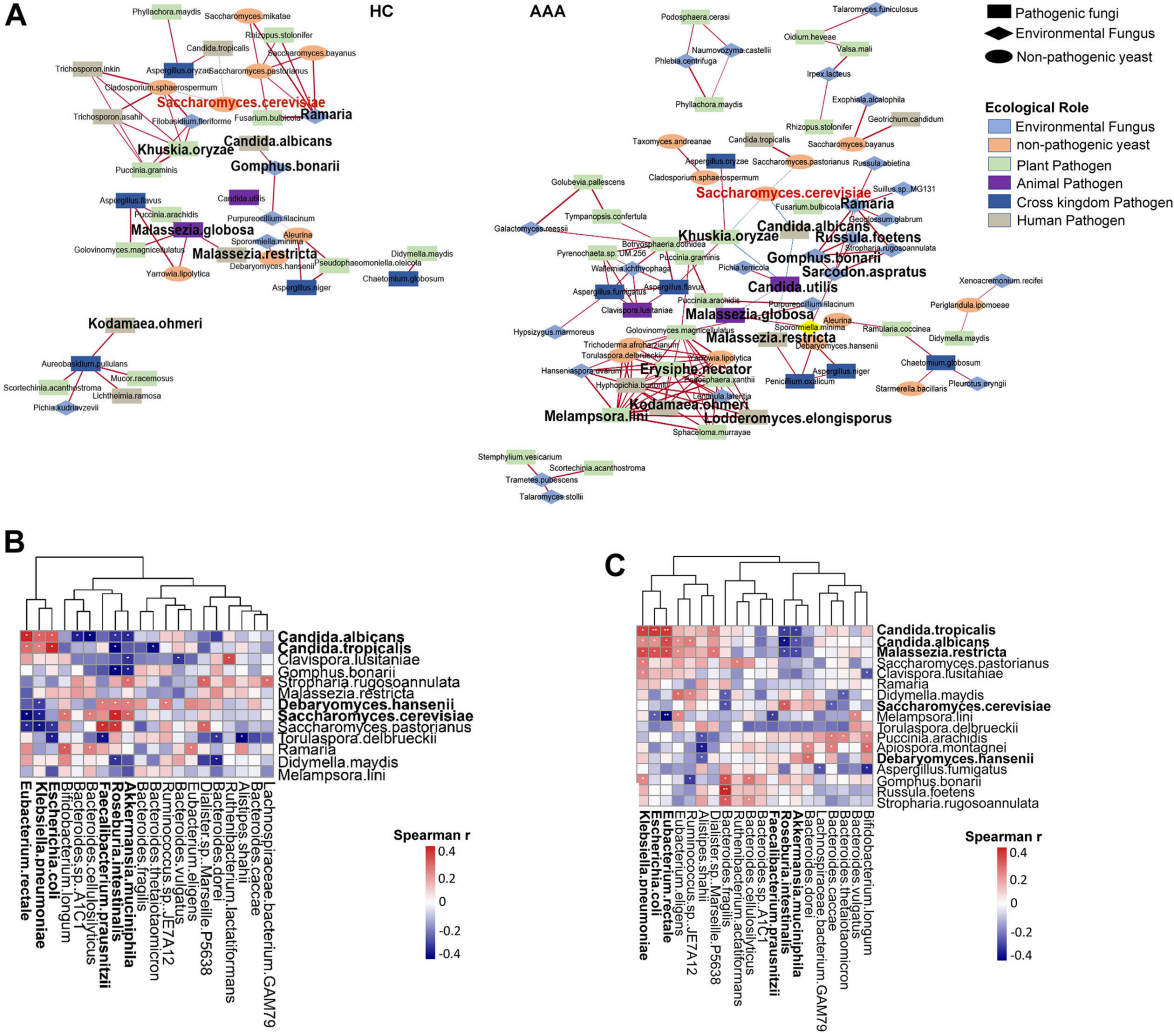
Network analysis of interactions among gut fungi and between fungi and bacteria. (**A, B**) Co-occurrence networks of abundant gut fungi in healthy individuals (HC) and AAA patients (AAA). The thickness of the edges represents the strength of correlations. Blue (red) edges indicate negative (positive) correlations between species. (**B, C**) Heatmap of correlations between intestinal fungi and bacteria in HC (**B**) and AAA (**C**) groups. Only significant correlations were shown (positive correlation: |r| > 0.6; negative correlation: |r| > 0.3; and FDR-adjusted *P* value < 0.05, FDR < 5%). Spearman’s correlation test with Benjamini-Hochberg adjustment was used. *P < 0.05; **P < 0.01.

Symbiotic network analysis at the species level of gut fungi revealed that the correlations among gut fungal species in both healthy individuals (HC) and AAA patients (AAA) were mainly positive. However, the co-occurrence rate of species with significant correlations between the two groups was only 25.6% (31/121), indicating that the symbiotic state of the gut fungal community is significantly altered in AAA. Compared to healthy individuals, correlations among gut fungal species in AAA patients were significantly more numerous and stronger. For example, the positive correlations between environmental fungi (such as *Ramaria*, *Russula foetens*, *Sarcodon aspratus*, and *Gomphus bonarii*) and multiple pathogenic fungi, including *Melampsora lini*, *Kodamaea ohmeri*, *Lodderomyces elongisporus*, and *Erysiphe necator*, were notably strengthened. In addition, the negative correlations between pathogenic fungi (*Malassezia globosa*, *Malassezia restricta*, *Khuskia oryzae*, and *Candida albicans*) and non-pathogenic yeasts (*Saccharomyces cerevisiae* and *Saccharomyces pastorianus*) were significantly strengthened. These results suggested that the symbiotic relationships between gut fungal species became more complex in AAA, which might be an adaptation to the specific intestinal microenvironment of AAA patients (Fig. 3A).

Furthermore, in healthy individuals, non-pathogenic yeasts such as *Saccharomyces cerevisiae* and *Debaryomyces hansenii* exhibited significant positive correlations with short-chain fatty acid (SCFA) producers or potential probiotics like *Roseburia intestinalis, Faecalibacterium prausnitzii*, and *Akkermansia muciniphila*, and negative correlations with harmful or opportunistic pathogens in the *Enterobacteriaceae* family, including *Escherichia coli*, *Klebsiella pneumoniae*, and *Eubacterium rectale*. By contrast, *Candida* species (*Candida albicans* and *Candida tropicalis*) showed trends opposite to those of non-pathogenic yeasts. However, in AAA patients, the correlations between fungi (*Saccharomyces cerevisiae* and *Debaryomyces hansenii*) and bacteria were reduced, while the correlations between pathogenic fungi (*Candida tropicalis*, *Candida albicans*, and *Malassezia restricta*) and harmful or opportunistic pathogens (*Escherichia coli*, *Klebsiella pneumoniae*, and *Eubacterium rectale*) were enhanced (Fig. 3B and C). These results suggested that the symbiotic network between gut fungi and bacteria in AAA patients was significantly changed, which might be closely related to AAA development.

### Diagnostic potential of gut mycobiome dysbiosis for AAA

**Fig 4:**
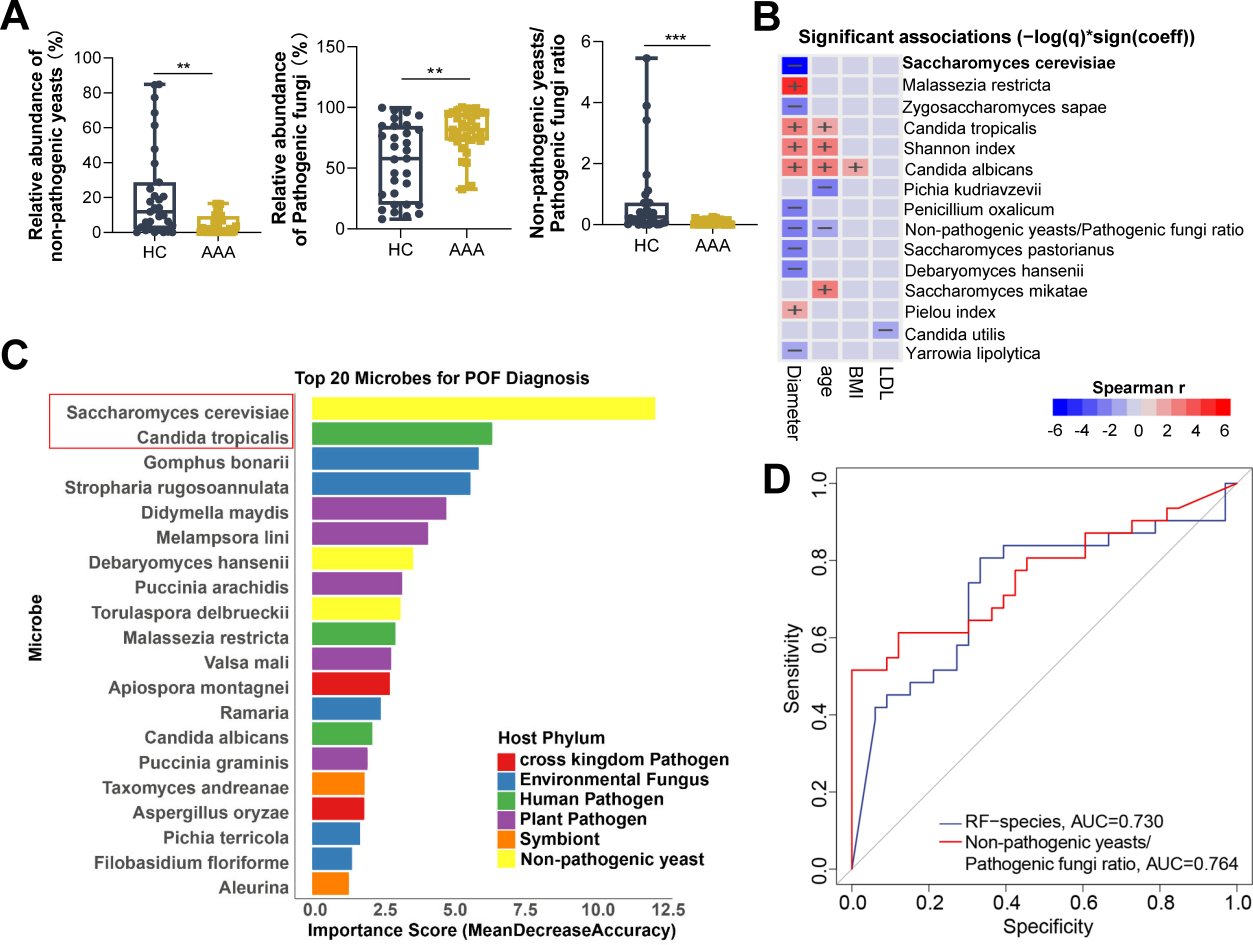
Diagnostic potential of gut mycobiome dysbiosis in AAA. (**A**) Comparison of the relative abundances of gut non-pathogenic yeasts (left panel), pathogenic fungi (middle panel), and their ratio (right panel) between healthy individuals (HC, *n* = 31) and AAA patients (AAA, *n* = 33). Two-tailed Wilcoxon rank-sum test was used. **P < 0.01; ***P < 0.001. (**B**) Maaslin2 analysis of associations between clinical parameters and fungal abundances at the species level, α-diversity indices, and the non-pathogenic yeasts-to-pathogenic fungi ratio. (**C, D**) Random Forest analysis of characteristic fungal species (**C**) and receiver operating characteristic (ROC) curves (**D**) showing the diagnostic potential of characteristic species and the non-pathogenic yeasts-to-pathogenic fungi ratio for AAA.

Our above results showed that the abundance of several pathogenic fungi and non-pathogenic yeasts in the gut of AAA patients was significantly altered, and they were significantly correlated with a variety of beneficial and harmful gut bacteria or opportunistic pathogens. Therefore, we further assessed the total abundance of pathogenic fungi and non-pathogenic yeasts in healthy individuals (HC) and AAA patients (AAA). The results showed that the abundance of non-pathogenic yeasts in the gut of AAA patients was significantly decreased, while the abundance of pathogenic fungi was significantly increased, leading to a significant reduction in the non-pathogenic yeasts-to-pathogenic fungi ratio (Fig. 4A). We then used Maaslin2 analysis to assess the correlation between these changes and clinical indicators. The results showed that the α diversity indices (Shannon and Pielou) were significantly positively correlated with AAA diameter. Pathogenic fungi, including *Candida albicans*, *Candida tropicalis*, and *Malassezia restricta*, were also significantly positively correlated with AAA diameter. In addition, *Candida albicans* was significantly positively correlated with age and BMI, and *Candida tropicalis* was significantly positively correlated with age. This is consistent with previous reports that *Candida* species tend to accumulate with age (23). Conversely, *Saccharomyces cerevisiae*, *Saccharomyces pastorianus*, *Debaryomyces hansenii*, and *Yarrowia lipolytica*, as well as the non-pathogenic yeasts-to-pathogenic fungi ratio, were significantly negatively correlated with AAA diameter. These results suggested that the gut mycobiome dysbiosis, characterized by significant changes in the abundance of pathogenic fungi (such as *Candida* and *Malassezia*) and non-pathogenic yeasts, was significantly correlated with the condition of AAA (Fig. 4B). To evaluate the diagnostic potential of these changes, we used random forest to identify the best biomarker combination, consisting of *Saccharomyces cerevisiae* and *Candida tropicalis*. We then performed ROC curve analysis using this combination and the non-pathogenic yeasts-to-pathogenic fungi ratio. The results showed that the biomarker combination and the ratio could effectively distinguish AAA patients from healthy individuals, suggesting that the gut mycobiome had great potential in the diagnosis of AAA (Fig. 4C and D).

### *Saccharomyces cerevisiae* significantly ameliorated the condition in AAA mice

**Fig 5:**
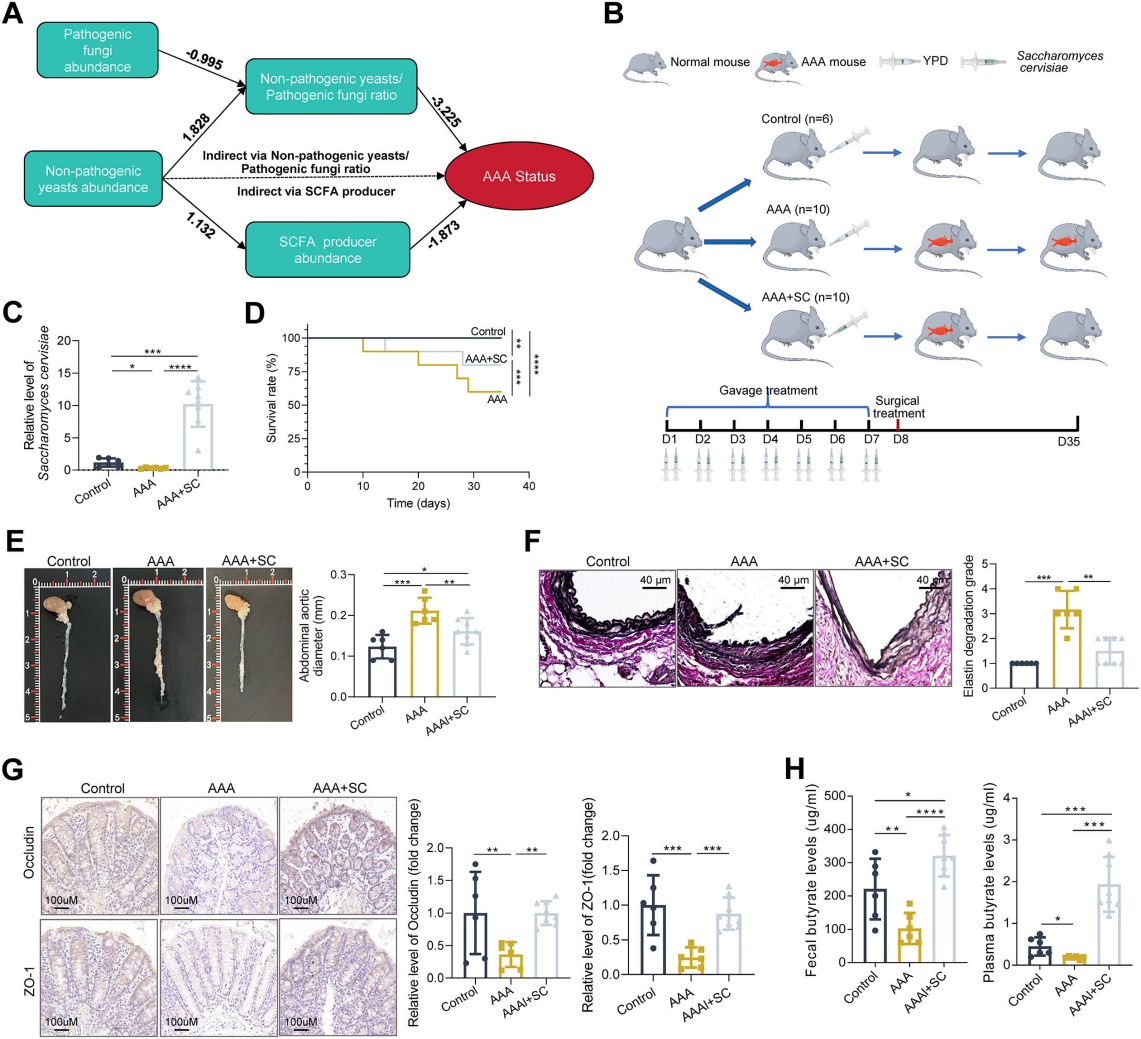
Effects of *Saccharomyces cerevisiae* on CaCl₂-induced AAA mice. (**A**) Causal pathway analysis of variables affecting AAA status, illustrating the causal relationships among pathogenic fungi, yeast, SCFA producers, non-pathogenic yeasts-to-pathogenic fungi ratio, and AAA status. Arrows indicates the direction of causal influence, and numbers represent standardized path coefficients. (**B**) Schematic diagram of the experimental procedure for evaluating the therapeutic effects of *Saccharomyces cerevisiae* on AAA. (**C**) Comparison of fecal levels of *Saccharomyces cerevisiae* among different groups of mice. (**D**) Kaplan-Meier survival curves showing “time-to-event” for each group of mice. (**E**) Representative images of abdominal aortic aneurysm dimensions (left panel) and quantitative analysis of aortic diameters in each group of mice (right panel). (**F**) Representative images of VVG staining of the abdominal aorta from the three groups of mice (left panel) and quantitative comparison of VVG staining (right panel). (**G**) Immunohistochemical staining showing the expression of intestinal barrier proteins (left panel) and their quantitative analysis (right panel) among the three groups. (**H**) Comparison of butyrate levels in feces and plasma of each group of mice. Control: sham surgery mice, *n* = 6; AAA: AAA mice induced by CaCl₂, *n* = 10; AAA + SC: AAA mice gavaged with *Saccharomyces cerevisiae*, *n* = 10. For C, E, F, and the left panel in G, one-way ANOVA followed by Fisher’s LSD post hoc test was used. For D, the log-rank (Mantel-Cox) test was used. For the right panel in G and H, a non-parametric Kruskal-Wallis test followed by a pairwise Wilcoxon rank-sum post hoc test was used. *P < 0.05; **P < 0.01; ***P < 0.001; ****P < 0.0001.

The above results suggested that the dysbiotic gut mycobiome, especially the significantly increased abundance of *Candida* and *Malassezia* and the significantly decreased abundance of non-pathogenic yeasts, was markedly correlated with the condition of AAA. However, research on the causal relationship between gut fungi and AAA, as well as the therapeutic potential of fungi in this context, remains limited. Structural equation modeling (SEM) analysis showed that the decrease in non-pathogenic yeasts and the increase in pathogenic fungi, leading to a reduced non-pathogenic yeasts-to-non-pathogenic ratio, affected AAA diameter. Non-pathogenic yeasts also influenced AAA *via* interactions with SCFA-producing bacteria. The abundance of most of these bacterial species was significantly reduced in AAA patients (Fig. 5A; Fig. S1A). To explore the therapeutic potential of non-pathogenic yeasts in AAA, AAA mice induced by CaCl₂ were gavaged with a *Saccharomyces cerevisiae* suspension (Fig. 5B). This yeast species exhibited the strongest negative correlation with aneurysm diameter and was significantly reduced in AAA patients (Fig. 4B; Fig. S1B). The treatment significantly increased the abundance of *Saccharomyces cerevisiae* in the gut of recipient mice (Fig. 5C) and prolonged their survival time (Fig. 5D). AAA mice displayed a significantly increased diameter of the abdominal aorta, marked disruption of the elastic lamina, and evident AAA formation, alongside notable damage to the intestinal mucosal barrier. However, *Saccharomyces cerevisiae* treatment significantly ameliorated these pathological changes in AAA mice (Fig. 5E through 5G). Furthermore, *Saccharomyces cerevisiae* treatment significantly increased the level of butyrate in the intestine and plasma of AAA mice (Fig. 5H). The findings indicated that *Saccharomyces cerevisiae* supplementation markedly ameliorated the condition in AAA mice, implying its therapeutic potential in managing AAA.

## DISCUSSION

In this study, we profiled the gut fungal community of healthy individuals and AAA patients using metagenomic sequencing. Our results revealed significant dysbiosis in the gut mycobiome of AAA patients, notably a marked reduction in the ratio of beneficial yeasts (primarily *Saccharomyces cerevisiae*) to pathogenic fungi (such as *Candida* and *Malassezia* species). We also highlighted the diagnostic potential of gut fungi in AAA and demonstrated the therapeutic efficacy of *Saccharomyces cerevisiae* in treating AAA. These findings deepen our understanding of gut microbiota imbalance in AAA and provide potential targets for novel therapeutic strategies.

While fungal detection in fecal samples commonly employs internal transcribed spacer (ITS) sequencing (25), our study utilized metagenomics to profile the gut mycobiome of AAA patients and healthy individuals. Traditional analytical methods have been shown to map approximately 0.01% of metagenomic data to fungal genomes. Using Kraken2 and Bowtie2, we extracted and annotated fungal-derived reads, achieving a mapping ratio of about 0.05% of intestinal fungal DNA. The fungal species composition revealed by our analysis largely aligned with those identified in large-scale studies, validating the successful construction of the gut mycobiome based on metagenomic data in our study. In AAA patients, α-diversity indices, including Shannon, Simpson, and Richness, were significantly increased, indicating greater complexity of the fungal communities and potential changes in the intestinal environment (26, 27). At the phylum level, AAA patients exhibited significantly higher abundances of *Basidiomycota* and notable reductions in *Ascomycota*, *Microsporidia*, and *Mucoromycota*. At the family level, the abundances of *Strophariaceae*, *Gomphaceae*, *Tricholomataceae*, *Didymellaceae*, *Ustilaginaceae*, *Porotheleaceae*, *Bertiaceae*, and *Cystobasidiaceae* were significantly enriched in AAA patients, while *Trichosporonaceae* was markedly decreased. At the genus level, the abundances of *Gomphus*, *Stropharia*, *Tricholoma*, *Didymella*, *Moesziomyces*, *Saccharomyces*, *Lactarius*, *Nakaseomyces*, and *Malassezia* significantly increased, whereas *Debaryomyces* and *Torulaspora* decreased substantially. In addition, some pathogenic fungi, including *Malassezia globosa*, *Malassezia restricta*, and *Candida albicans*, showed enhanced correlations with other fungi, suggesting that abnormal inter-fungal relationships were the important characteristics of AAA-associated gut fungal dysbiosis. In terms of bacterial-fungal correlations, AAA patients exhibited strengthened positive correlations between various pathogenic fungi and harmful bacteria. Conversely, the positive correlations between non-pathogenic yeasts and beneficial bacteria, as well as negative correlations between non-pathogenic yeasts and harmful bacteria, were attenuated in AAA patients.

AAA is characterized by its critical and life-threatening nature (28), with its etiology remaining poorly understood (29, 30). Some studies have demonstrated significant changes in the gut mycobiome in various diseases, including colorectal cancer (CRC) and chronic heart failure, suggesting that gut fungi may be closely associated with the development of these diseases (31, 32). In our study, we revealed significant differences in the gut microbial composition of AAA patients compared to healthy controls. In AAA patients, the proportion of non-pathogenic yeasts and the ratio of non-pathogenic yeasts to pathogenic fungi were significantly lower, while the proportion of pathogenic fungi was markedly higher than that in healthy individuals. This pronounced imbalance was associated with clinical indicators such as aneurysm diameter and age. These findings suggested that the non-pathogenic yeasts-to-pathogenic fungi ratio could serve as a biomarker for monitoring AAA progression. Causal analysis using SEM indicated that the reduction of intestinal yeasts might contribute to AAA progression. Furthermore, our animal experiments using the AAA mouse model also demonstrated the causal relationship between the dysbiotic gut mycobiome and AAA development. These findings indicated that the dysbiotic gut mycobiome was an important pathological characteristic of AAA patients and might play a significant role in the development of AAA.

The fungi like *Candida albicans* have been reported to modulate immune responses, influencing inflammation and contributing to various diseases like IBD (33). Our study found a significant increase in *Candida albicans* and *Malassezia* species (*Malassezia restricta* and *Malassezia globos*a) in AAA patients, suggesting these fungi might promote AAA development. *Candida albicans* is known to exacerbate cardiovascular diseases by enhancing gut permeability and systemic inflammation, while *Malassezia* species, typically skin resident, have been linked to chronic gut inflammation (34). By contrast, the abundance of *Saccharomyces cerevisiae*, a beneficial fungus known for its role in maintaining the gut barrier and suppressing inflammation (35, 36), was significantly reduced in AAA patients. Our animal experiments showed that its supplementation delayed AAA progression. These findings highlight the complex role of gut fungi in AAA progression, where the proliferation of pathogenic fungi and the reduction of beneficial fungi may jointly drive AAA development. Modulating the gut fungal community could offer new strategies for AAA prevention and treatment.

Until now, AAA treatment remains a significant challenge, particularly for patients ineligible for surgical intervention (37). Gut microbiota-based therapies offer a promising approach for slowing AAA progression. Our previous work demonstrated the protective role of *Roseburia intestinalis* in AAA, mediated by its production of butyrate (38). In the present study, we identified the reduction of *Saccharomyces cerevisiae* as an important contributor to AAA development. Therefore, we evaluated the potential of *Saccharomyces cerevisiae* as a therapeutic strategy for treating AAA by administering it *via* gavage to AAA mice induced by CaCl_2_. Our results demonstrated that *Saccharomyces cerevisiae* produced multiple beneficial effects in AAA mice, including significantly prolonged survival, reduced aneurysm diameter, diminished damage to the elastic laminae of the abdominal aorta, and marked amelioration of intestinal mucosal barrier injury. These findings provide compelling evidence for the protective role of *Saccharomyces cerevisiae* in AAA, supporting its potential as a novel therapeutic target for AAA management.

Interactions among gut microbiota, particularly between fungi and bacteria, are crucial for intestinal homeostasis and disease processes. Fungal-bacterial competition and metabolic cooperation, such as utilizing each other’s metabolites like SCFAs, help maintain a healthy gut environment (39). SCFAs have been demonstrated protective effects on cardiovascular diseases by suppressing inflammatory responses and improving vascular function (40). As an important product of microbial fermentation, butyrate plays crucial roles in regulating immune responses, maintaining intestinal barrier function, and suppressing inflammation, thereby delaying the progression of cardiovascular diseases (41). Our previous studies also demonstrated that *Roseburia intestinalis* alleviated AAA by producing butyrate (38). Yeasts have been reported to alter the metabolic activities of gut bacteria through interactions, leading to increased butyrate production (42). In this study, we identified, for the first time, a significant positive correlation between *Saccharomyces cerevisiae* and SCFA producers in the human gut, particularly the butyrate-producing *Roseburia intestinalis*. Moreover, we observed a significant elevation in butyrate levels in the serum and feces of *Saccharomyces cerevisiae*-treated AAA mice, suggesting this may be a potential mechanism for the protective effect of *Saccharomyces cerevisiae* against AAA. In addition, we found that in AAA patients, the abundance of pathogenic fungi, such as *Candida*, significantly increased, contrasting with the marked decrease in non-pathogenic yeasts. This pathogenic overgrowth may disrupt the gut microbial balance, increase inflammation, and consequently promote AAA development (43).

While this study offers promising insights into the therapeutic potential of yeast in AAA treatment, several limitations must be acknowledged. First, the relatively small sample size restricts the generalizability of the findings. Second, since the experiments specifically targeted certain yeast types (e.g., *Saccharomyces cerevisiae*), the results may apply to other yeast species. Significant physiological and metabolic variations exist among different yeasts, potentially affecting their efficacy in similar applications (44). Moreover, further investigation into the molecular mechanisms by which yeasts mitigate AAA progression is essential. Identifying key metabolites remains a critical area for future research. Our analysis revealed that in AAA patients, the reduced yeast population primarily originated from dietary sources, highlighting diet as a key factor in regulating intestinal fungi. However, we did not collect detailed dietary information from participants and could not conduct further research on this aspect.

In conclusion, this study characterized the gut mycobiome profiles of healthy individuals and AAA patients, identifying a significant imbalance between non-pathogenic yeasts and pathogenic fungi in AAA patients. Furthermore, animal experiments confirmed the therapeutic potential of *Saccharomyces cerevisiae* in mitigating AAA progression. Our findings deepen the understanding of the role of gut fungi in AAA and provide compelling evidence for yeast-based AAA treatment. Although gut fungal research is still in its infancy compared to gut bacterial studies, it holds immense potential for clinical applications and warrants further investigation.

## MATERIALS AND METHODS

### Study design, subject recruitment, and sample collection

In all, 33 patients with AAA were recruited from Qilu Hospital of Shandong University. All patients were diagnosed with unruptured AAA by computed tomography angiography (CTA) (45). In all, 31 healthy controls were recruited from the Health Examination Center of Qilu Hospital and were confirmed to be free of AAA by abdominal CT or MRI. The demographic and clinical characteristics of the healthy volunteers were matched to those of the AAA patients. Inclusion criteria were first diagnosis and AAA diameter ≥3 cm. Exclusion criteria included age >80 years, severe hypertension, severe diabetes, severe dyslipidemia, etc. Samples were collected on the morning of the second day after admission, with fresh fecal and plasma samples stored at −80°C.

### AAA mouse model

Male C57BL/6J mice (6- to 8-week old) were purchased from SPF Biotechnology Co., Ltd. (Beijing, China) and housed under SPF conditions at 23±1°C, 50%–60% humidity, with a 12-hour light/dark cycle, and fed standard chow and autoclaved water. An AAA model was induced using CaCl₂ one week after fungal transplantation (46). Mice were anesthetized with intraperitoneal sodium pentobarbital, a midline abdominal incision was made, and a 0.5M CaCl₂-soaked cotton ball was placed for 20 minutes, followed by a PBS-soaked cotton ball for 10 minutes. The sham control group was treated with 0.9% NaCl. After 28 days, mice were euthanized, and tissues were collected.

### Fecal genomic DNA extraction

Genomic DNA was extracted from 200 mg fecal samples using 2 × CTAB buffer and phenol-chloroform as described previously (31). The DNA was purified with a DNA column and eluted in sterile Milli-Q water, and its integrity was assessed by formaldehyde agarose gel electrophoresis. DNA concentration and purity were measured using a NanoDrop 2000 (Thermo Scientific).

### Shotgun metagenomic sequencing and nonredundant gene catalog construction

Raw sequencing data underwent quality control using FastQC (v 0.11.9) (47) and the removal of the adapter and low-quality read with Trimmomatic (v 0.39) (48). Kraken2 (v 2.1.2) (49) and GTDB 207 (50) were employed to filter bacterial sequences. Fungal species were identified through alignment to a reference fungal genome using Bowtie2 (v 2.4.4) (51). The number of effective sequences for fungal identification was determined as previously described and the catalog annotation of fungal species was performed using FungalTraits (52).

### Diversity analysis and network visualization

α-diversity metrics, including Shannon, Simpson, Richness, and Pielou indices, were calculated using USEARCH to assess the richness, and evenness of fungal communities within each sample. β-diversity was evaluated using Bray-Curtis dissimilarity and PCoA. Co-occurrence networks based on fungal taxa co-occurrence frequency were constructed and visualized using Gephi.

### Analysis of butyrate levels

To quantify butyrate levels, samples were homogenized and treated with 0.5% phosphoric acid. Following centrifugation, the upper organic phase was extracted using ethyl acetate. After adding an internal standard, butyrate concentrations were determined using an Agilent 7890A-5975C GC-MS system.

### Fungi and fungal transplantation

The *Saccharomyces cerevisiae* AH22 strain (ATCC 38626) was purchased from the American Type Culture Collection and cultured in YPD medium at 37°C with shaking at 150 rpm. Mice were randomly divided into three groups: Control, AAA, and AAA + SC group. Control mice received 0.9% NaCl instead of 0.5 M CaCl₂ during the AAA model establishment procedure. All mice were gavaged daily with 200 µL YPD medium for 7 days before surgery. AAA group mice continued receiving YPD medium, while AAA + SC group mice were gavaged daily with 200 µL *Saccharomyces cerevisiae* at 1 × 10^8^ CFU/mL for 7 days before AAA model induction. Fecal DNA was extracted from mouse droppings and analyzed by qPCR to quantify *Saccharomyces cerevisiae* DNA levels.

### Real-time quantitative PCR

Total RNA was extracted from samples using TRIzol and reverse-transcribed to cDNA using the PrimeScript RT Kit (Takara, Kusatsu, Japan). Genomic DNA was extracted from fecal samples using a modified CTAB method. PCR amplification was performed using TB Green Premix Ex Taq and specific primers, and PCR products were detected using a LightCycler 480 system (Roche, Basel, Switzerland). Relative gene expression was calculated using the 2^−ΔΔCt^ method. A complete list of all primers used in this study is provided in Table S2.

### Immunohistochemistry and Verhoeff-Van Gieson staining

Mouse tissues were fixed in 4% paraformaldehyde, dehydrated through graded ethanol, embedded in paraffin, and sectioned at 5 µm thickness. Sections underwent antigen retrieval in tris-EDTA buffer (pH = 9) and were blocked with 5% BSA. They were then incubated with primary and HRP-conjugated secondary antibodies (Table S3). Visualization was performed using DAB (ZSGB-BIO, ZLI-9018) and hematoxylin staining. VVG staining was conducted following the manufacturer’s instructions (Abcam, ab150667). The stained sections were scanned using a Pannoramic SCAN II scanner (3DHistech, Budapest, Hungary) to acquire digital images.

### Statistical analysis

Data normality was assessed by the Shapiro–Wilk test and homogeneity of variances was tested with Levene’s test. For comparison between the two groups, data that passed both tests were analyzed using a two-tailed Student’s *t*-test. Data that passed the Shapiro–Wilk test but not Levene’s test were analyzed using Welch’s *t*-test, while data failing the Shapiro–Wilk test were compared using a two-tailed Wilcoxon rank-sum test. For comparison among multiple groups, data meeting both normality and homogeneity assumptions were analyzed using one-way ANOVA with Fisher’s least significant difference (LSD) post hoc test. Data meeting the normality assumption but not homogeneity were analyzed using Welch’s ANOVA with the Games–Howell post hoc test. Non-normally distributed data were assessed using the Kruskal–Wallis test with pairwise Wilcoxon rank-sum post hoc tests. Intergroup separation trends in PCA and PCoA were evaluated using Adonis for PERMANOVA or ANOSIM for rank-based group differences. Spearman correlations and corresponding *P*-values were calculated using the corr.test function in the “psych” R package, with *P*-values adjusted for multiple comparisons using the Benjamini–Hochberg or Holm–Bonferroni method. All statistical analyses were performed using R (v 4.2.0) and GraphPad Prism (v 9.4.1).

## Data Availability

The metagenomic sequencing data used in this study were previously generated and publicly available in the NGDC database under the accession number PRJCA011493. The original study should be cited accordingly (DOI: 10.1016/j.chom.2022.09.004).

## SUPPLEMENTAL MATERIAL

Supplemental material spectrum.01776-24-s0001.docx Tables S1 to S3; Fig. S1.

ASM does not own the copyrights to Supplemental Material that may be linked to, or accessed through, an article. The authors have granted ASM a non-exclusive, world-wide license to publish the Supplemental Material files. Please contact the corresponding author directly for reuse.
