# Supplementary material for: The role of dysbiotic gut mycobiota in modulating risk for abdominal aortic aneurysm
Source: Microbiol Spectr. 2024 Sep 24;12(11):e01776-24. doi: 10.1128/spectrum.01776-24 (PMC11537029; doi:10.1128/spectrum.01776-24)
Supplement: Supplemental material — Tables S1 to S3; Fig. S1. [file spectrum.01776-24-s0001.docx]

**SUPPLEMENTAL INFORMATION**

**Table S1. Clinical data of healthy individuals and AAA patients**

| **Characteristics** | **Healthy (n = 31)** | **AAA (n = 33)** | **Adjusted P values** |
| --- | --- | --- | --- |
| Age (year) | 67.77 ± 5.04 | 68.73 ± 7.13 | 0.5 |
| BMI (kg/m^2^) | 23.41 ± 2.25 | 24.56 ± 2.51 | 0.26 |
| SBP (mmHg) | 138.03 ± 15.79 | 147.39 ± 12.37 | 0.12 |
| DBP (mmHg) | 81.03 ± 9.16 | 84.12 ± 8.72 | 0.28 |
| TG (mmol/L) | 1.24 ± 0.61 | 1.35 ± 0.73 | 0.57 |
| TC (mmol/L) | 4.53 ± 0.86 | 4.14 ± 1.23 | 0.25 |
| HDL (mmol/L) | 1.39 ± 0.35 | 1.20 ± 0.41 | 0.12 |
| LDL (mmol/L) | 2.72 ± 0.72 | 2.56 ± 0.83 | 0.41 |
| **AAA diameter (cm)** | - | **6.47 ± 2.62** | - |

**Note:** BMI, Body Mass Index; SBP, Systolic Blood Pressure; DBP, Diastolic Blood Pressure; TG, Triglycerides; TC, Total Cholesterol; HDL, High-Density Lipoprotein; LDL, Low-Density Lipoprotein.

**Table S2. Primers used in this study for qPCR experiments.**

| **Target species** | **Forward Primer** | **Reverse Primer** |
| --- | --- | --- |
| *Roseburia intestinalis* | GCGGTRCGGCAAGTCTGA | CCTCCGACACTCTAGTMCGAC |
| *Faecalibacterium prausnitzii* | CCCGGCATCGGGTAGAG | GGACGCGAGGCCATCTC |
| *Eubacterium rectale* | CGGTACCTGACTAAGAAGC | AGTTGATTCTTGGTGGAAC |
| *Roseburia hominis* | CCCACTGACAGAGTATGTAATGTAC | GCACCACCTGTCACCAC |
| *Akkermansia muciniphila* | CAGCACGTGAAGGTGGGGAC | CCTTGCGGTTGGCTTCAGAT |
| *Bifidobacterium bifidum* | TACGAGATTTGAGCCACTGT | CGCTGGCAACACAAATCATC |
| *Lactobacillus acidophilus* | CATCCAGTGCAAACCTAAAG | GATCCGCTTGCCTTCGCA |
| *Saccharomyces cerevisiae* | CCACCTCACCACTTCCAACT | GGAGCGCTAGGGTAAGCATA |
| *Bacteria general* | CGGTGAATACGTTCCTGG | CGGTGAATACGTTCCTGG |

**Table S3. Antibodies used in this study.**

| **Antibodies** | **Source** | **Cat No.** |
| --- | --- | --- |
| rabbit anti-Occludin | proteintech | 13409-1-AP |
| rabbit anti-ZO-1 | BOSTER | PB9234 |
| goat anti-rabbit | abcom | ab6721 |

**Supplemental Figure**

**
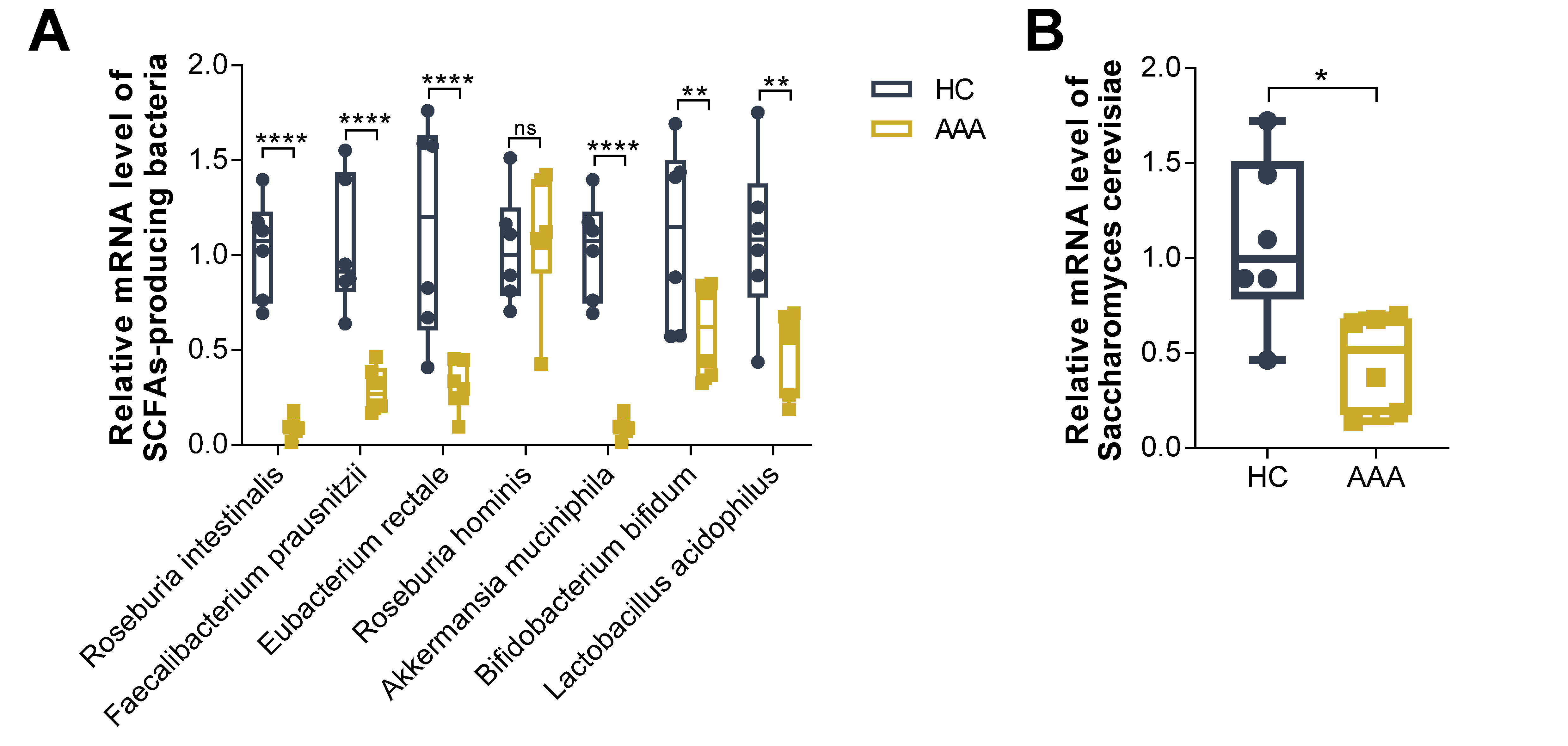
**

**Figure S1.** **Abundance changes in SCFAs-producing bacteria and *S. cerevisiae* in the gut of AAA Patients. (A)** Comparison of the abundance of SCFAs-producing bacteria detected by qPCR in healthy individuals (HC) and AAA patients (AAA), n=6. **(B)** Comparison of the abundance of *Saccharomyces cerevisiae* detected by qPCR in HC and AAA groups, n=6. Significant was determined using a Two-tailed Wilcoxon rank-sum test. ns: not significant; *: p < 0.05; **: p < 0.01; ****: p < 0.0001.
